# Postgraduate radiology education in Nigeria: Looking backward and forward

**DOI:** 10.4102/sajr.v22i1.1362

**Published:** 2018-08-29

**Authors:** Bukunmi M. Idowu

**Affiliations:** 1 Union Diagnostics and Clinical Services, Lagos, Nigeria

## Abstract

Formal postgraduate radiology education (residency training) in Nigeria commenced in 1976 at the University College Hospital, Ibadan, Oyo State. Currently, two postgraduate medical colleges (one national and the other international or regional) are saddled with the responsibility of superintending the training programme. This is a chronicle of the evolution of radiology in Nigeria with emphasis on the current status of the training programme and areas that require improvement. Though the programme has delivered a quality healthcare workforce for the country and the international community since inception, significant but surmountable difficulties still exist. It is hoped that all stakeholders and policy-makers will take note and safeguard the future of the specialty.

## Introduction

Nigeria is the most populous African nation with an estimated population of 191 million people^1^ in 2017 and is located in West Africa between latitudes 4°N and 14°N, and longitudes 2° E and 15° E. There are 36 states or provinces, a federal capital territory and 774 local council areas in the country.

Postgraduate medical education has been defined as the ‘constellation of learning activities carried out to enable doctors to develop relevant competencies and deeper knowledge in specific subject areas after completion of basic medical education’. It encompasses ‘pre-registration training, vocational and/or professional training, specialist and subspecialist training, as well as other forms of training obtained after the initial undergraduate medical education’ with the aim of ‘developing new knowledge and innovations for high-level medical practice’.^2^ The all-encompassing training serves to prepare them for future roles as clinicians, mentors, educators, researchers and administrators.^3^ Until the establishment of postgraduate medical colleges in Nigeria, Nigerian medical specialists were trained abroad, mainly in England, Scotland, Ireland and the United states of America (US).

Establishment of a local training programme has been shown to be more sustainable and cost-effective than reliance on expatriates.^3,4^ It may also help to stem the emigration of highly-skilled physicians (the brain drain menace).^5,6^ In addition, training in the local environment would enable trainees to provide much needed clinical service delivery simultaneously.^7^

As previously observed:

… there is a lack of literature about the radiology education infrastructure in Africa. This gap in the literature can be challenging for radiologists who would like to collaborate, contribute, and learn from differences, similarities, and challenges in radiology education systems outside their countries.^8(p. 1)^

This article attempts to chronicle the evolution of postgraduate radiology education in Nigeria, identify existing impediments to the training of world-class radiologists, proffer solutions and make recommendations to facilitate improvement.

## Brief history of radiology in Nigeria

The first X-ray machine was installed in the first radiology department in Nigeria at the Lagos General Hospital, Lagos State, in 1913.^4^ Angiography and fluoroscopy machines were installed at the University College Hospital (UCH), Ibadan, Oyo State, Southwest Nigeria, in 1961 and 1972, respectively.^9^ The first ultrasound machine in Nigeria was also installed in UCH in 1975.^4,10^ Installation of the first computed tomography (CT) scanner, also in UCH Ibadan, followed much later in 1987 and remained the only CT scanner in the country until 1995.^4,11^ The first magnetic resonance imaging (MRI) machine in Nigeria was commissioned at the National Hospital, Abuja, in 1999.^4^ Since these landmark events, radiography machines and ultrasound scanners have become ubiquitous in the country. Multislice CT scanners have also become more available in the urban centres (public and private hospitals and diagnostic centres); in fact, a state-of-the-art Toshiba Aquilon 640-slice CT scanner was installed at the Ibom Specialist Hospital, Akwa-Ibom state, in 2015. There are now 1.5 Tesla MRI machines in an increasing number of private and a few public health facilities across the country.

The first generation of Nigerian radiologists who trained in the United Kingdom obtained their diploma in medical radiodiagnosis (DMRD) from the Royal Colleges of Physicians and Surgeons of London, United Kingdom, while some obtained additional fellowship subsequently. The first Nigerian to qualify as a radiologist was Dr Michael Aneke Benedict Ogakwu (DMRD 1960).^4^ He was followed by others in radiodiagnosis in that decade (Table 1). The first Nigerian professor of radiology was Prof S.B. Lagundoye who was appointed on 01 October 1976.^9^ Prof B.C. Umerah also became a professor in 1977. The first Nigerian woman to qualify as a radiologist was Dr Foluso A. Ladapo-Elebute (DMRD 1971)^4^ followed by the other pioneer women mentioned in Table 2. Dr Ladapo-Elebute was also the first Nigerian female professor of radiology. The first American-trained Nigerian radiologist was Dr John Chukwudi Odita who became a diplomate of the American Board of Radiology in 1975 and a board-certified paediatric radiologist in 1995.^4^ Dr Taiyewo Moses Kolawole was the first Nigerian to obtain a fellowship (FFR) in general radiology and was also the first subspecialist, having completed a fellowship in neuroradiology at the University of Western Ontario in 1977.^12^

**TABLE 1:** Pioneer radiologists in Nigeria.

No	Name	DMRD	FFR or FRCR	FMCR	FWACS
1	Dr Micheal Aneke Benedict Ogakwu	1960	DU	1970	1976
2	Dr Adebayo Olumide Banjo	1962	1995	1970	1976
3	Dr Henry Ebenezer I. Innis-Palmer	1963	DU	1978	DU
4	Dr Ekpo Edet Eyo†	1963	DU	DU	DU
5	Dr Adeyemi Oladipo Olowu	1964	DU	1982	DU
6	Dr Edwin Gbefememre Ogidi	1964	DU	DU	DU
7	Prof. Suleiman Botsende Lagundoye	1966	1993	1970	1978
8	Prof. Taiyewo Moses Kolawole	1968	1970	1981	1976
9	Prof. Benjamin Chukwunyelu Umerah	1969	1973	1981	1982
10	Dr Gabriel Adetayo Akinhanmi	1970	DU	1981	1979
11	Prof. Rafiu Akinsanya Alamu Obisesan	1970	1974	1978	1976
12	Dr B.A.O. Udoakang	1970	DU	DU	DU

*Source*: Based on the nominal rolls or registers of fellows (West African College of Surgeons and National Postgraduate Medical College of Nigeria) and Nzeh DA, Lagundoye SB, editors. Devices and images: 50 years history of the Association of Radiologists of West Africa (1962–2012).

S/No, number; DU, data unavailable; DMRD, diploma in Medical Radiodiagnosis of the Royal Colleges of Physicians and Surgeons of London; FFR, fellowship of the Faculty of Radiology of the Royal Colleges of Physicians and Surgeons of London which transmuted into FRCR in 1980 when the faculty obtained a royal Charter; FRCR, fellowship of the Royal College of Radiologists; FMCR, fellowship of the Nigerian Medical Council in Radiology which is now fellowship of the National Postgraduate Medical College of Nigeria (Radiology); FWACS, fellowship of the West African College of Surgeons (Radiology).

† , He was a specialist physician and a radiologist having first obtained the membership diploma of the Royal College of Physicians (MRCP) in 1960.

**TABLE 2:** Pioneer women radiologists in Nigeria.

No	Name	DMRD	FFR or FRCR	FMCR	FWACS
1	Prof. Foluso A. Ladapo-Elebute	1971	DU	1981	1984
2	Dr Georgiette Olufemi Williams	1972	DU	1981	1987
3	Dr Mercy Idemokuna Ojemuyiwa	1973	DU	1981	1982
4	Dr F.O. Sijuwola	1974	DU	DU	DU
5	Prof. Dorothy I. Makanjuola	1975	1977	1981	1982
6	Dr Omolara Mojisola Roberts	1975	DU	1981	1982
7	Dr Irene Ebisan Rewane	1975	1977	1981	DU
8	Dr Iyabode Yetunde Osinaike	1978	DU	1982	1997
9	Dr Enitan Bolut Odutola	1979	DU	DU	1985
10	Dr Felicia Temitayo Soneye-Vaughan	DU	DU	1981	1983

*Source*: Based on the nominal rolls or registers of fellows (West African College of Surgeons and National Postgraduate Medical College of Nigeria) and Nzeh DA, Lagundoye SB, editors. Devices and images: 50 years history of the Association of Radiologists of West Africa (1962–2012).

S/No, number; DU, data unavailable; DMRD, diploma in Medical Radiodiagnosis of the Royal Colleges of Physicians and Surgeons of London; FFR, fellowship of the Faculty of Radiology of the Royal Colleges of Physicians and Surgeons of London which transmuted into FRCR in 1980 when the faculty obtained a royal Charter; FRCR, fellowship of the Royal College of Radiologists; FMCR, fellowship of the Nigerian Medical Council in Radiology which is now fellowship of the National Postgraduate Medical College of Nigeria (Radiology); FWACS, fellowship of the West African College of Surgeons (Radiology).

There are two professional bodies of radiologists in the country: a regional association (Association of Radiologists of West Africa [ARAWA]) founded in 1962 and a national association (Association of Radiologists in Nigeria [ARIN]) which was incorporated and registered with the Corporate Affairs Commission in 2010. These two bodies have a healthy working relationship, with ARAWA being responsible for regional cooperation and integration in the west African sub-region, while ARIN is responsible for handling national issues within Nigeria. Nigerian radiologists can belong to both bodies simultaneously. Member countries of ARAWA currently include anglophone West African countries (Nigeria, Ghana, Liberia, Sierra-Leone and The Gambia), after the francophone countries effectively pulled out of ARAWA in 1989 by forming the Societe de Radiologie d’Afrique Noire Francophone (Radiological Society for Francophone Africa).^4^

The two postgraduate medical colleges saddled with the responsibility of training radiologists (and other medical specialists) locally, are the West African College of Surgeons (WACS) and the National Postgraduate Medical College of Nigeria (NPMCN). The WACS was inaugurated in 1960 as the Association of Surgeons of West Africa which morphed into WACS in December 1972.^4,13^ The National Postgraduate Medical College of Nigeria started as the postgraduate programme of the Nigerian Medical Council or the National Medical College in 1970 and segued into the NPMCN in 1979.^2,7^ Overseas-trained fellows constituted the founding fellows of the radiology faculty in both colleges, while other foreign trained fellows who qualified subsequently became fellows of the colleges or radiology faculty by election. These colleges function separately though they cooperate with each other. The West African College of Surgeons was set up by the heads of government of the aforementioned five anglophone West African countries, while the Federal Government of Nigeria set up the NPMCN. Both are meant to produce a medical specialist workforce to meet the healthcare needs of the respective localities.

The NPMCN Faculty of Radiology had 21 foundation fellows (this includes few radiotherapists).^2^ Formal radiology residency training by NPMCN commenced in 1976 with four candidates (Dr Obadiah Folorunsho Komolafe, Dr Ayotunde Oluremi Ogunseyinde, Dr Solomon Sunday Daini and Dr Adebayo Olanrewaju Taiwo).^4^ Dr O.F. Komolafe was inducted as the first fellow by examination of the NPMCN Faculty of Radiology in May 1980. As of December 2011, the NPMCN had produced a total of 145 radiologists by examination.^4^

The radiology faculty was one of the six founding faculties of WACS. Dr Henry Peter Adebayo Adedokun was the first to obtain the WACS radiology fellowship by examination in 1983.^4^ The next set of WACS radiology fellows qualified in October 1984: Col (Dr) A.I. Audu, Dr Kolawole Olufemi Iyun and Dr Johnny Uzoma Valmon Monu.^13^ As of December 2011, WACS Faculty of Radiology had produced a total of 210 radiologists by examination.^4^ It must be noted that a number of these radiologists hold the fellowships of both colleges.

It is noteworthy that between 1960 and 1980 when the country produced the first locally trained radiologist, only about 40 radiologists (two per annum) were successfully trained abroad.^4^ As of 2015, an estimated 250–300 radiologists practise in the country at a ratio of one radiologist to 566 000 people.^14^

## Eligibility for postgraduate radiology education in Nigeria

Undergraduate medical education in Nigeria lasts a minimum of six years for students who gain admission via the Unified Tertiary Matriculation Examination (UTME) route or a minimum of five years for those admitted via the direct entry route (undertaken by candidates with advanced-level certificates or those who already have first degrees in medical-related courses). Successful medical students are awarded the MBBS or MBChB or MBBCh degree on completion of undergraduate studies. There are 32 fully accredited and six partially accredited medical schools in Nigeria with a capacity to train a total of 3325 medical doctors annually.^15^ The new doctors are granted temporary registration with the Medical and Dental Council of Nigeria (MDCN), allowing them to practise under the supervision of an attending or consultant. Full registration is obtained upon successful completion of a compulsory 1-year housemanship or internship in an accredited training centre. After internship, those still younger than 30 years of age proceed on a compulsory 1-year National Youth Service Corp (NYSC) scheme. Thereafter, a doctor becomes eligible to attempt the primary (qualifying) examinations of the postgraduate medical colleges.

The first (1976) set of NPMCN radiology residents sat the primary examination. Thereafter, a waiver was granted by both colleges in order to attract young doctors to the ‘unpopular’ specialty. The waiver had to be nullified after more than two decades when the number of doctors applying for radiology residency exceeded the available training slots. Consequently, NPMCN recommenced primary examinations in September 2001 (three candidates, one passed), while WACS started theirs in October 2004. In essence, there were no radiology primary exams between 1976 and 2001 or 2004. The two postgraduate faculties of radiology organise the primary examination for prospective residents biannually. The result of the primary exams enjoys reciprocal validity between the two colleges after payment of an administrative (exemption) fee.

The primary examination (‘primaries’) comprises multiple-choice questions in three subjects: basic physics, anatomy and clinical medicine. It is, in addition to a hospital-based interview, a prerequisite for admission into a formal radiology residency training programme at accredited hospitals across the country. The hospital-based pre-admission written and/or oral interviews are conducted independently by each training centre and are held on average about once in two years. The intake per hospital varies – usually, less than 20% of those who apply are admitted (in some cases, less than 5%). The hospitals train the residents, while the postgraduate colleges conduct the periodic examinations and award the fellowships.

## Current structure of radiology residency in Nigeria

The radiology faculties of both postgraduate colleges are responsible for supervision of radiology residency, curriculum design and periodic review, accreditation of training centres and conducting three examinations biannually (primary examination, part one [membership] examination and part two [fellowship] examination). The admission requirements for both colleges are similar but there are some (minor) differences in the curricula and structure of their training programmes.

Categories of residents in training include primary residents (employed and paid by the hospital in which they are undergoing residency training), supernumerary residents (sponsored by another institution to the hospital in which they are doing their residency training) and self-sponsored residents.

Currently, 13 teaching hospitals have full accreditation, while eight have partial accreditation of the NPMCN for radiology residency training. Of these 21, only four are owned by state or provincial governments, while the others are owned by the federal government of Nigeria.^16^ Fourteen training centres have full accreditation of the WACS radiology faculty, while two have partial accreditation.^17^ Some of the training centres have concurrent accreditation from both colleges.

The purpose of the accreditation is to: ‘ensure that minimum standards in faculties and staffing are maintained in training institutions to facilitate training to the level expected’.^18^ The accreditation process evaluates the prospective training centres based on the following criteria^19,20^ that are scored quantitatively: qualifications and experience of personnel and faculty (prescribed number, appropriate trainers: trainees ratio, support staff); availability of infrastructure (electricity, water, radiological suites, call rooms); availability of core and support equipment; structure and content of the training programme (didactic lectures, tutorials, read-out sessions, and so on); opportunities for or evidence of skill acquisition (procedure register, log book); access to new information (Internet access, departmental library) and regular feedback or evaluation (periodic continuous assessment, annual progress report). The recommended ratio of resident doctors to consultants or attendings is a minimum of 3:1 or maximum 4:1, that is, one senior registrar and two registrars or two senior registrars and two registrars to one consultant or attending. Trainers and examiners are required to be at least five years post-fellowship. The colleges determine the number of training slots allotted to each training centre based on the number of trainers (one trainer: four trainees) and availability of the aforementioned training facilities. If a training centre can expand and provide more of these requirements, then the colleges could allow them more training slots.

Full accreditation (all the requirements for accreditation are satisfied) lasts for 5 years, while partial accreditation (only some of the requirements for accreditation are satisfied) lasts for 2 years before another review or reassessment. A centre may get partial accreditation if one of the core radiological equipment (CT, MRI and fluoroscopy) is faulty or unavailable or if the trainees far outnumber the trainers. Resident doctors from centres with partial accreditation have to undergo training in the deficient area in one of those with full accreditation (external posting) before becoming eligible for examination. A resident doctor can undergo the radiology training programmes of both colleges simultaneously. Failure of a resident to pass the part one or part two examinations after three sittings or failure to complete the entire programme within the stipulated five years (depending on the policy of each training centre) may lead to withdrawal from the radiology residency training programme. Such withdrawn (relocated) candidates can attach themselves to another fully accredited training centre to complete their training. However, if a candidate is relocated before passing part one, he or she reverts to medical officer status. Both colleges organise separate part one and part two examinations which do not enjoy reciprocity unlike the primary examinations.

Success in the part one examination is a pre-requisite to membership of either college. In order to be eligible for this exam, residents (registrars) must have undergone at least 24 months of actively supervised radiology rotations evidenced by in-training assessment scores and completed logbooks. The rotations or postings include dark room, plain radiography, fluoroscopy, mammography, ultrasonography, CT and MRI. The order of rotation differs amongst the training centres. In addition, candidates must have attended update or revision courses in radiological or medical physics and clinical radiology which are organised by the colleges. The part 1 examination comprises multiple-choice questions (medical physics, radiological anatomy, clinical radiology), essays (clinical radiology), image slideshows (radiological anatomy, clinical radiology) and image reporting sessions or viva voce. Successful residents are upgraded and admitted into the senior residency training programmes as senior registrars. A senior resident is required to rotate through the same radiology postings (except dark room and medical physics) for another 24 months, conduct a radiological research and submit a dissertation. In addition to these, the NPMCN requires the submission of a book of at least six case reports that the candidate managed during their period of training.

Success in the part two examination admits the senior resident into the fellowship of the college whose exam they passed.^21^ The part two comprises clinical multiple-choice questions, essays (WACS only), slideshow, image reporting sessions or viva voce and defence of the candidate’s dissertation. Dissertation defence has been a component of NPMCN radiology part two examinations, since the first fellow of the faculty by examination sat the examination in 1979. In contrast, dissertation defence for WACS radiology part two examinations commenced in October 2009, having been waived hitherto alongside the primary examination at the inception of the WACS radiology faculty. Regarding the part two dissertation, after passing the part one exam, the candidate chooses a topic (guided by their departmental supervisor and policy) and submits a proposal to the college, after obtaining study approval from the ethics committee of the training institution (two different proposals are submitted separately if the candidate wishes to pursue the part two programmes at both colleges). On receiving the proposals (and payment of assessment fees), the faculty secretary and/or chairman selects two external assessors (similar to journal peer reviewers) outside the training centre of the candidate to vet the submitted proposal. The reports of the assessors determine whether the proposal is accepted or rejected by the faculty. If rejected, the candidate restarts the process with a new topic or substantial modification of the proposal. If accepted, the candidate may proceed with the study. Both outcomes are often communicated to the candidate in writing. To be eligible for NPMCN part 2 exams, the proposal must have been approved at least 12 months prior to the time of screening application forms, log books and completed dissertations for the exams. During the part two exams, the candidate faces the hitherto anonymous external assessors to defend their dissertation. There are three possible outcomes of the dissertation defence: full (straight) pass, provisional pass (need to make minor corrections) and failed defence (major flaws rendering the dissertation unacceptable with a need to rewrite the dissertation or reconduct the study and resit the defence in six months or one year).

## Problems and challenges

### General problems with residency training in Nigeria

Despite its successes, a myriad of problems confront postgraduate medical education in Nigeria. Previous authors^2,22,23,24,25,26,27^ have written extensively about these but key impediments will be highlighted here.

Residency training in Nigeria has no legal imprimatur. Indeed, the entire training programme has been whimsically suspended twice in the last five years by the federal government. Only recently has a bill been submitted to parliament to secure legal backing and access to annual budgetary provisions. Currently, the programme is poorly funded and curriculum review is not regular.

There is a shortage of trainers and lack of commitment by trainers. A previous study^22^ put the contribution of trainers to the training of residents in Nigeria at 26% – 50%. Trainees often complain of being subjected to various forms of bullying.^28,29^ Furthermore, the trainers do not undergo performance appraisal by their trainees. In addition, an endemic patronage culture means that competent hands are sometimes omitted in the selection of trainers or academics for the training centres. Similarly, the system of recruiting examiners for the postgraduate colleges needs to be more transparent.

The current over-emphasis on service provision rather than training is not helpful. With resident doctors working between 80 and 168 h per week, the programme appears to be managed as a conduit for cheap assistants and workforce to the training centres or trainers rather than an educational enterprise.^2,22^

The fellowships versus PhD controversy introduced another dimension into postgraduate medical education in Nigeria. In 2008, the National Universities Commission (NUC) decreed that those who do not possess a PhD can no longer teach at universities.^2,26,30,31,32^ The issue has lingered and is yet to be resolved conclusively, leaving much uncertainty in the minds of resident doctors and junior consultants. Presently, there are no PhD programmes in radiology in Nigerian universities though some specialties are already developing the curriculum.

Research proposals submitted by residents to the colleges for approval suffer needless delays without consequent sanction of the offending examiners. This often increases the duration of training for affected residents, exposing them to the risk of expulsion from the programme before completion.

There are other sundry issues, including inadequate training centres and training slots, incessant industrial disharmony in the health sector which often disrupts training,^33,34^ the penchant of the WACS to charge exorbitant or prohibitive fees for its services and the abysmal pass rate in college exams.^35^

Brain drain is also hitting the programme hard – the few specialists being trained are emigrating in droves to Europe, the Caribbean, US, Canada, Australia, the Middle East and even other African countries, lured by better remuneration, living and working conditions and a quest to raise their children in an environment with better opportunities. Others leave because of the rather paradoxical lack of employment opportunities after completing their training – this may be due partly to the problematic 2010 agreement between the academic staff union of universities (ASUU) and the federal government to raise the retirement age of Professors from 60 to 70 years. Consequently, vacancies for academic positions are not as readily available as before.

### Problems peculiar to radiology residency training

Some problems are peculiar to radiology residency, partly because of constantly evolving technological advancement.^8,14,36,37,38,39^ The curriculum for radiology residency is designed to produce general radiologists. There is no formal subspecialty training in Nigeria at the moment, although there is a growing interest amongst residents and consultants in the commencement of subspecialty training.^36,37^ This is as a result of the availability of more sophisticated imaging modalities in private and some public hospitals and a higher demand for more qualitative reports by clinicians.

Conventional radiography and ultrasound are the only regularly functioning modalities in many training centres often because of frequent breakdown of equipment without prompt repair. Computed tomography, MRI, fluoroscopy and mammography units are the worst affected. In fact, the fluoroscopy units in the vast majority of training centres are out of service. This sad reality forces many residents to choose ultrasound-based topics for their part two dissertations.

The perennial problem of epileptic power supply in the country has had a pernicious effect on radiology training. It is often responsible for equipment breakdown and most of the training centres resort to running their equipment on generators which adds to operative costs and is often difficult to sustain.

Some modern training equipment or facilities are unavailable in some of the training centres – picture archive computerised system (PACS), electronic reporting systems, planar scintigraphy, positron emission tomography (PET) scanners, elastography, breast tomosynthesis, interventional radiology and transcranial Doppler. The lack of PACS means that several interesting teaching cases are lost irretrievably. Many of the available CT scanners in the training centres are four slices with only a few centres having 64-slice or 128-slice scanners. Similarly, the MRI scanners in most of the training centres are low-field strength (0.2–0.3 Tesla) units.^38^ These low-tech machines often produce comparably low-quality images.

Finally, instruction in medical physics is still suboptimal and this perennial problem has plagued the programme since inception.^4,40^ There have been improvements of late but they need to be sustained.

## Recommendations

From the foregoing, it is clear that radiology residency training in Nigeria needs to be strengthened in order to continue delivering on its core mandate areas of training and research. In addition to the general proposals for improving postgraduate medical education in Nigeria (comprehensive structural or organisational review, improved funding, curriculum review, retraining of trainers and incorporation of regular appraisal or monitoring or evaluation),^2^ the following would help to improve radiology training in particular:

Commencement of subspecialty training in the country is necessary.^36^ The Society for Paediatric Imaging in Nigeria (SPIN)^41^ and the Nigeria Society for Interventional Radiology (NiSIR) have already been formed but are still in its infancy. Other subspecialties need to take root in the country as quickly as possible to make the programme more robust.

Bidirectional performance appraisal where trainees also get to evaluate their trainers is overdue.^42^ The current system suffers from little or no accountability on the part of the trainers.^43^

Furthermore, addition of leadership training and non-interpretive skills (quality improvement, patient safety, professionalism and ethics, compliance or regulatory or legal issues, research and imaging informatics) to the radiology residency curriculum is highly desirable.^44,45,46,47^

Re-instatement of the overseas clinical attachment and/or getting external examiners from the Royal College (UK) or American board (USA) to partake in the fellowship examinations could help give the programme a greater international outlook and acceptability. The format of the fellowship exams of both NPMCN and WACS could also be aligned to the Royal College exam pattern as has been successfully implemented in various forms by Malaysia, Hong Kong, South Africa^48^ and Singapore.^49^

Intercalation of PhD and fellowship programmes as has been conducted in the USA^50^ or a modification of the South African Master of Medicine programme^48^ would seem a logical way out of the needless controversy over possession of PhD by medical doctors at universities.^51^

Institution of departmental equipment budget for the servicing and replacement of old equipment, equipment donation by philanthropists and public–private partnership schemes for the procurement and maintenance of equipment will help in running the training centres sustainably.^52^

The process of employment into academic or trainer positions in the training centres needs to be more merit-driven. The current system is unduly influenced by politics of various shades, primordial sentiments, patronage and personal idiosyncrasies.

In addition to professional qualifications and competencies, there is a need to instruct trainers in modern medical education and pedagogical techniques.^29,53^ Undergoing the fellowship training of the Foundation for Advancement of International Medical Education and Research (FAIMER) is recommended.^54^

Finally, the author agrees fully with the submission of Rabinowitz et al.^39(p. 7)^ that:

Radiologists, radiology organizations, and radiology vendors in high-income countries are in a unique position to help African training programs, with donations of money, teaching time, teaching materials, equipment, and equipment service contracts. It is important that such endeavours be undertaken with knowledge of local political and medical systems to avoid wasting scarce, donated resources.

Fellowship and observership opportunities are also invaluable.

## Conclusion

There is no doubt that the radiology residency programme in Nigeria has reasonably fulfilled its purpose since inception. Training the next generation of radiologist physicians and trainers is an important task that requires meticulous planning, absolute commitment and adequate funding. The programme needs to be streamlined and properly repositioned to keep producing competent radiologists who are capable of fitting into the future roles of radiology in healthcare delivery.^55^
